# Comparative Study of Cold Sensation, Pinprick, and Perfusion Index in Evaluating the Quality of Ultrasound-Guided Supraclavicular Brachial Plexus Block: An Observational Study

**DOI:** 10.7759/cureus.80211

**Published:** 2025-03-07

**Authors:** Rakesh Bahadur Singh, Atit Kumar, Amit K Singh, Vikram S Rathore, Aman Kumar

**Affiliations:** 1 Anesthesiology, Uttar Pradesh University of Medical Sciences, Etawah, IND; 2 Anesthesiology and Critical Care, Uttar Pradesh University of Medical Sciences, Etawah, IND

**Keywords:** cold sensation, perfusion index, pin prick, scbpb, ultrasound-guided supraclavicular brachial plexus block

## Abstract

Background

Ultrasound (USG)-guided brachial plexus block has become the preferred method for surgeries of the upper limb as it reduces the risks of pneumothorax, nerve injury, and local anesthetic toxicity. An objective, noninvasive technique for evaluating the block's effectiveness is the perfusion index (PI). An increased PI suggests a successful brachial plexus block.

Aims and objectives

The study aimed to evaluate and compare cold sensation, pinprick, and PI to determine the most effective indicator for assessing the quality of USG-guided supraclavicular brachial plexus blocks (SCBPBs).

Materials and methods

The study included 92 patients. Each patient received 25 mL of local anesthetics (10 mL of 0.5% bupivacaine, 10 mL of 2% lignocaine, and 5 mL of 0.9% normal saline) for USG-guided SCBPB. Responses to cold sensation, pinprick, and PI were noted for operative and control limbs starting right after block introduction till 20 minutes at intervals of 5 minutes. After 20 minutes, a modified Bromage scale was used to evaluate the motor block, and a 3-point scale was used to evaluate the block's quality. If the block quality was unsatisfactory or resulted in complete failure, such patients were considered for general anesthesia.

Results

Significantly higher PI in the blocked arm than the unblocked arm was observed across all periods. The PI and PI ratio cut-off values at 10 minutes were 3.25 and 2.75, respectively. At 10 minutes, the PI's sensitivity, specificity, and negative predictive value (NPV) were 100%. At 10 minutes, the sensitivity of the pinprick and cold sensation was 75% and 82%, respectively, and at 20 minutes, it reached 100%. All three methods' positive predictive value was 100% at 10 minutes. The NPV for cold sensation and pinprick gradually increased, reaching 100% at 20 minutes. Area under the receiver operating characteristic curve (AUROC) analysis showed that the PI reached a value of 0.983 by 10 minutes.

Conclusion

PI values were significantly higher in the blocked arm than in the unblocked arm. A successful block can be predicted with 100% sensitivity and specificity at 10 minutes if the PI is more than 3.25 and the PI ratio is more than 2.75. The AUROC analysis confirms that PI values are reliable early indicators of block success.

## Introduction

The brachial plexus is an intricate network of nerves that extends from the neck to the axilla, providing motor and sensory fibers to the upper limbs. The supraclavicular brachial plexus block (SCBPB) is a widely employed anesthetic technique for upper extremity surgeries, known for its efficacy in providing anesthesia and analgesia [1]. When the supraclavicular technique is used to block this plexus, it can successfully block the musculocutaneous and ulnar nerves, which may be missed with other techniques such as the axillary or interscalene methods. However, due to the pleura near the vicinity, many anesthesiologists hesitate to attempt the supraclavicular approach. Ultrasound (USG)-guided techniques minimize the risk of pneumothorax, speed up the onset of the block, and reduce the amount of local anesthetic needed [2].

The success of this block depends on using accurate assessment methods to evaluate sensory and motor function after the procedure. Traditional evaluation techniques, such as cold sensation and pinprick tests, ensure sufficient anesthetic coverage. However, these methods can be subjective and vary between assessors [3].

Newer and more objective approaches are based on assessing the increased blood flow, local vasodilation, and increased skin temperature in the blocked region following local anesthetic injection [4]. Recent advancements have introduced the perfusion index (PI) as an objective, quantifiable measure of the quality of nerve blocks and their success rates [5].

The photoplethysmographic signal on pulse oximetry is used to calculate the ratio of pulsatile to non-pulsatile blood flow, or PI. It indicates the blood flow rate and correlates strongly with nerve block efficacy. The PI is a non-invasive marker of peripheral perfusion at the sensor site and an index for sympathetic blockade [6]. The PI ratio is the ratio of PI in the blocked arm or control arm at 10 and 0 minutes.

The primary objective of this study was to compare the efficacy of the PI with traditional assessment methods, cold sensation and pinprick, in evaluating the quality of the USG-guided SCBPB. The secondary objective of the study is to determine the most appropriate cutoff values for the PI that can reliably indicate a successful anesthetic outcome. These findings could optimize anesthetic practices and improve patient safety by reducing the risk of inadequate anesthesia. By ensuring that practitioners have access to reliable and effective tools to assess the quality of regional anesthesia, we can ultimately enhance patient outcomes in upper extremity surgeries.

## Materials and methods

This prospective observational study was conducted following approval from the institutional ethical committee (ethical clearance number: 46/2022-23, dated 22/12/2022). The trial was registered with the Clinical Trial Registry of India (CTRI/2023/12/060785). The study was conducted between December 2023 and October 2024 at the Uttar Pradesh University of Medical Sciences, Etawah, Uttar Pradesh, India. For elective upper limb surgery, 92 adult patients between 18 and 65 years of age and having American Society of Anesthesiologists physical status I-II were included in the study. Patient refusal, proximal humerus and shoulder surgery, allergy to local anesthetic, infection at the injection site, extensive burn wounds in either arm, diabetes mellitus, and peripheral vascular disease were excluded from the study.

Sample size calculation

The sample size was calculated using the following formula [7]:

 $n=(Z^2(&prop;&frasl;2)&times;V(AUC))/d^2)$

In this formula, n = sample size, Zα/2 = value from the standard normal distribution reflecting the confidence level, i.e., 1.96 at a 95% confidence level, and d signifies the margin of error, set at 0.075, which constitutes 10% of the area under the curve (AUC). The variance of the area under the curve, V(AUC), was calculated using the expression:

 $𝑉(𝐴𝑈𝐶) = (0.0099 &times; 𝑒^{𝑎^{2}/2}) &times; (6𝑎^{2} + 16)$

$𝑉(𝐴𝑈𝐶) =0.1347$

where a=ϕ^-1^(AUC)×1.414 and ϕ^-1^ is the inverse of the standard cumulative normal distribution. By substituting an AUC value of 0.75 into the calculations, the resulting minimum sample size was determined to be 92.

Anesthesia administration procedure

A comprehensive history was taken, and meticulous physical and systemic examinations were performed on all patients before surgery. All study participants tendered written and informed consent before participation. All 92 patients were instructed to abstain from oral intake for 8 hours before surgery. The night before surgery, 150 mg of ranitidine and 0.5 mg of alprazolam tablets were given to each patient as premedication. In the operating room, emergency drugs and airway management equipment were arranged. The intravenous line was established using an 18- or 20-gauge cannula and primed with 10 mL/kg of Ringer's lactate solution before administering the block. The patient was connected to ECG leads, a pulse oximeter, and a non-invasive blood pressure monitor. Mean arterial pressure, systolic and diastolic blood pressure, pulse rate, respiration rate, and preoperative baseline oxygen saturation were measured.

All SCBPBs were conducted under stringent aseptic conditions by senior anesthesiologists proficient in USG-guided nerve blocks. The USG equipment (Sonosite M-turbo USG machine, Bothell, WA, United States) with a high-frequency (6-13 MHz) linear array probe was used throughout the procedures. The probe was placed just above the middle of the clavicle in a coronal oblique plane. To get a cross-sectional picture of the pulsing subclavian artery, the transducer was angled toward the caudal end. Next to and below it were the linear hyperechoic structures of the first rib and the parietal pleura. The brachial plexus is identified as a dense aggregation of nerves, characterized by hypoechoic circular structures, positioned laterally and in proximity to the subclavian artery. The Stimuplex block needle (B Braun), 22 gauge, 50 mm, was inserted in a plane directed toward the brachial plexus, from lateral to medial. Following meticulous aspiration, 2 mL of local anesthetic was administered to confirm accurate needle placement. Subsequently, a total of 25 mL of local anesthetic was injected using the same needle, comprising 10 mL of 0.5% bupivacaine, 10 mL of 2% lignocaine, and 5 mL of 0.9% normal saline. Additional needle repositioning was performed when injecting local anesthetic, which did not seem to cause distribution in and around the brachial plexus.

Block success assessment

PI was noted in the operative and control limb at t = 0 (right after block). The patient's response to cold sensations and pinpricks was assessed in both limbs. The cold sensation was measured on both arms using a two-point scale: 0 (cold) and 1 (not cold). The pinprick sensation was checked using a 22-gauge hypodermic needle. The patient's reaction was recorded on a two-point scale: 0 (sensation) and 1 (no sensation). All three methods were repeated at 5-minute intervals on both limbs until 20 minutes.

The motor block was measured using the Modified Bromage scale [8], where 0 denoted normal motor function, i.e., full extension and flexion of the elbow, wrist, and fingers; 1 denoted diminished motor strength, i.e., the ability to move only the fingers; and 2 denoted a complete motor block, or the inability to move the elbow, wrist, and fingers.

The quality of the block was made based on a 3-point scale: 0 (complete failure), 1 (unsatisfactory), and 2 (satisfactory).

If the patient experienced discomfort at the site of surgery, the block was considered to have failed, and the patient was either given more analgesia or switched to general anesthesia, depending on their needs. Data of these patients were included for analysis in the demographic data; however, they were excluded from further study.

Statistical analysis

The mean and standard deviation were used to express the quantitative parameters. Frequency and percentage were used to express categorical data. The receiver operator characteristic (ROC) curves at different time intervals were plotted to measure the diagnostic accuracy of three methods - cold sensation, pinprick, and PI - in predicting the success of USG-guided SCBPB. Area under the receiver operating characteristic curve (AUROC) is an effective way to measure the relative accuracy of these methods. Cut-off values were calculated when PI can reliably signify a successful anesthetic outcome. P-values of <0.05 were considered significant, and P-values of <0.01 were considered highly significant. Relative performance of the three methods was also established by calculating sensitivity, specificity, positive predictive value (PPV), and negative predictive value (NPV). The data was entered in an MS Excel spreadsheet and analyzed using Python (v3.9) and its libraries, namely Numpy (v1.24), Pandas (v1.3.5), and Seaborn (v0.11.2).

## Results

This study was conducted to explore the efficacy of the PI compared to traditional assessment methods such as cold sensation and pinprick tests in assessing the success of SCBPB. A description of the demographic characteristics of all study participants is shown in Table 1. Out of 92 study participants, three had failed blocks. Comparisons of PI in both blocked and unblocked arms at different time intervals are shown in Table 2.

**Table 1:** Demographic characteristics of study participants (n=92) ASA PS, American Society of Anesthesiologists Physical Status; BMI, body mass index; SD, standard deviation

Demographic characteristics	Statistics value
Age (years)	42.35 ± 13.12
Gender (male:female)	16:7
BMI (kg/m^2^)	21.66 ± 1.28
Weight (kg)	62 ± 9.46
ASA PS (I:II)	15:8
Block final status (success:failure)	89:3

**Table 2:** Comparison of PI in blocked and unblocked arms at different time intervals *A p-value of <0.001 is considered highly significant. PI, perfusion index

Time interval (minutes)	PI in the blocked arm (mean ± SD)	PI in the unblocked arm (mean ± SD)	p-Value
0 minutes (just after block)	1.04 ± 0.16	0.94 ± 0.12	0.412
5 minutes	2.35 ± 0.4	1.05 ± 0.15	<0.001^*^
10 minutes	2.86 ± 0.49	1.21 ± 0.17	<0.001^*^
15 minutes	4.64 ± 0.83	1.02 ± 0.15	<0.001^*^
20 minutes	6.95 ± 1.24	1.14 ± 0.17	<0.001^*^

At 10 minutes, the PI ratios were 2.75 in the blocked arm and 1.29 in the unblocked arm, which was highly significant (p<0.001).

In Table 3, the relative performance of PI is evaluated against traditional methods of cold sensation and pinprick by calculating sensitivity, PPV, specificity, and NPV at different time intervals.

**Table 3:** Variation of sensitivity, specificity, PPV, and NPV variation for cold sensation, pinprick, and PI methods at different time intervals NPV, negative predictive value; PI, perfusion index; PPV, positive predictive value

Time (in minutes)	Traditional method and PI	Sensitivity	PPV	Specificity	NPV
0 minutes	Cold sensation	0	0	1	0.03
	Pinprick	0	0	1	0.03
	PI	0	0	1	0.03
5 minutes	Cold sensation	0	0	1	0.03
	Pinprick	0	0	1	0.03
	PI	0.74	1	1	0.12
10 minutes	Cold sensation	0.75	0.99	1	0.12
	Pinprick	0.82	1	1	0.16
	PI	1	0.99	1	1
15 minutes	Cold sensation	0.93	0.99	0.67	0.25
	Pinprick	0.97	1	0.64	0.5
	PI	1	0.99	1	1
20 minutes	Cold sensation	1	0.99	0.67	1
	Pinprick	1	0.99	0.67	1
	PI	1	0.99	1	1

**Figure 1:**
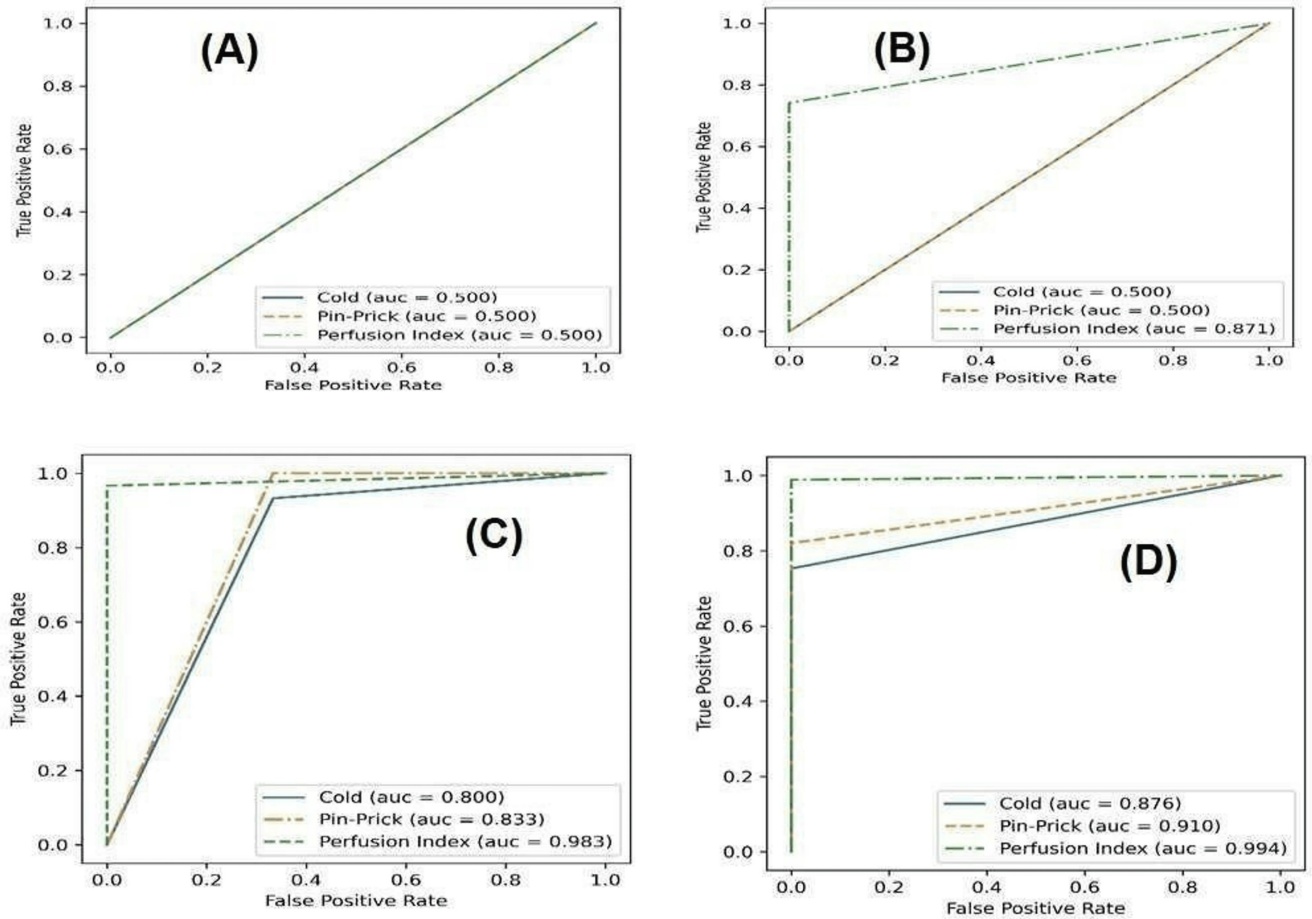
ROC curves and AUROC for cold, pinprick, and PI methods blocks at different time intervals: (A) at 0 minutes, (B) at 5 minutes, (C) at 10 minutes, (D) at 15 and 20 minutes. AUC, area under the curve; AUROC, area under the receiver operating characteristic curve; ROC, receiver operating characteristic

Relative performance of three methods - cold sensation, pinprick, and PI - in accurately predicting the success of a block is established by computing and comparing the AUROC in ROC curves at different time intervals, as shown in Figure 1.

**Figure 2:**
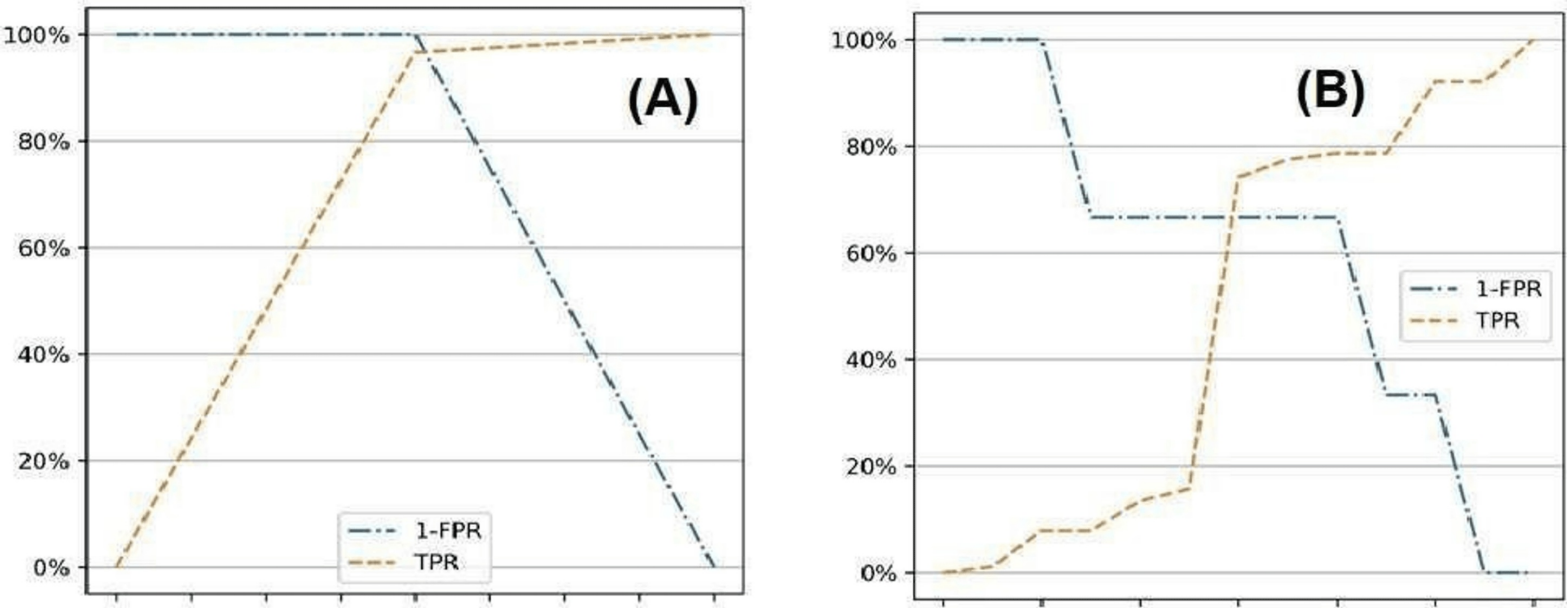
Cut-off calculation plots for PI ratio and PI at 10 minutes: (A) PI and (B) PI ratio. FPR, false-positive rate; PI, perfusion index; TPR, true-positive rate

To ensure that PI can be reliably used to establish the anesthetic outcome, the most suitable cut-off values are established (Figure 2). For PI at 10 minutes, the cut-off value came out as 3.25 and for PI ratio as 2.75. The cut-off value is at the point of intersection of TPR and (1-FPR).

## Discussion

USG-guided brachial plexus blocks (BPBs) have significantly enhanced the efficacy and precision of regional anesthesia by improving the accuracy of needle placement, reducing the risk of complications such as pneumothorax, nerve injury, and intravascular local anesthetic toxicity, and ensuring more effective pain management during upper extremity surgeries.

In our study, PI values increased significantly with time in the blocked arm compared to the unblocked arm. In their studies, Lal et al. [9], Veena et al. [10], and Kim et al. [11] recorded similar observations.

In this investigation, the PI and PI ratio cut-off values at 10 minutes were determined to be 3.25 and 2.75, respectively. Abdelnasser et al. [5] reported cut-off values as 3.3 and 1.4 for PI and PI ratio, respectively. Kim et al. [11] observed a cut-off value of 3.3 for the PI ratio in epinephrine group. Şeyhanlı et al. [12] observed the cut-off value of PI as 3.8. These observations in different studies are comparable to our study. Cut-off values observed by Rajesh et al. [13] were 5.1 and 4.7, which were significantly higher as compared to our study. This difference may be due to the use of different drugs at different concentrations at different sites.

In our study, the relative performance of PI is evaluated against traditional cold sensation and pinprick methods by calculating sensitivity, PPV, specificity, and NPV at different time intervals. Cold sensation, sensitivity, and PPV reached close to 100% by 20 minutes. NPV increased to 100% after 15 minutes, and specificity reduced from 100% to 67% by 20 minutes. Galvin et al. [14] observed that sensitivity and PPV never reached more than 60% and 68%, respectively, even after 30 minutes. Specificity and NPV also increased rapidly.

In our study, the pinprick method performed slightly better than the cold sensation for sensitivity, PPV, and NPV. However, it remained similar for specificity. Nallam et al. [15] observed loss of sensation at around 12.8 ± 3.8 in group LD50 (0.5% levobupivacaine and 50 μg of dexmedetomidine) and 8.2 ± 1.7 minutes in group LD100 (0.5% levobupivacaine and 50 μg of dexmedetomidine). Galvin et al. [14] observed sensitivity, and PPV never reached more than 80% and 63%, respectively, even after 30 minutes. Specificity and NPV also increased rapidly.

In this study, AUROC for cold sensation remained at 0.5 for 5 minutes. It increased to 0.80 at 10 minutes and stabilized to 0.876 after 15 minutes. AUROC for pinprick performed similarly to cold sensation, though slightly better after 10 minutes. Galvin et al. [14] observed that AUROC increased for cold sensation from 0.50 to 0.60 at 10 minutes and then to 0.63 at 15 minutes and to 0.75 at 20 minutes. For pinprick, the AUROC increased from 0.50 to 0.53 at 5 minutes and then to 0.68 at 10 minutes and to 0.78 at 20 minutes. This finding is very similar to that of this study. AUROC for PI in this study significantly improved to 0.871 at 5 minutes and stabilized to 0.994 at 15 minutes.

Limitations

This study has certain limitations. This study was conducted at a single institution. A multi-institutional study should be conducted, which will offer a wider diversity of patients. In addition, a blinded study can help eliminate any biases. Also, the follow-up period in this study was limited to 20 minutes. A study with a longer follow-up period can offer insight into any delayed variation of PI. Other studies have shown varying cut-off values, which calls for studies to evaluate the effective value of PI with different configurations of drugs, concentrations, and sites.

## Conclusions

PI values significantly increased in the blocked arm as compared to the unblocked arm. Cut-off values for PI ratio and PI were 2.75 and 3.25, respectively. With 100% sensitivity and specificity at 10 minutes, these can be employed to predict block success. AUROC analysis clearly outlines that PI values can be used as early, reliable, and objective indicators to predict the successful block. The findings of this study were reinforced by similar findings of other studies. Thus, PI value is a reliable and effective tool for evaluating the success of USG-guided BPB in comparison to traditional assessment by cold sensation and pinprick.
